# Insects attracted to Maple Sap: Observations from Prince Edward Island, Canada

**DOI:** 10.3897/zookeys.51.478

**Published:** 2010-07-23

**Authors:** Christopher G. Majka

**Affiliations:** 1 Research Associate, Nova Scotia Museum, 1747 Summer Street, Halifax, Nova Scotia, Canada, B3H 3A6

**Keywords:** Coleoptera, Nitidulidae, Lepidoptera, Xylenini, Trichoptera, Prince Edward Island, Canada, maple sap, maple syrup, biodiversity

## Abstract

The collection of maple sap for the production of maple syrup is a large commercial enterprise in Canada and the United States. In Canada, which produces 85% of the world’s supply, it has an annual value of over $168 million CAD. Over 38 million trees are tapped annually, 6.5% of which use traditional buckets for sap collection. These buckets attract significant numbers of insects. Despite this, there has been very little investigation of the scale of this phenomenon and the composition of insects that are attracted to this nutrient source. The present paper reports the results of a preliminary study conducted on Prince Edward Island, Canada. Twenty-eight species of Coleoptera, Lepidoptera, and Trichoptera were found in maple sap buckets, 19 of which are known to be attracted to saps and nectars. The physiological role of sap feeding is discussed with reference to moths of the tribe Xylenini, which are active throughout the winter, and are well documented as species that feed on sap flows. Additionally, 18 of the 28 species found in this study are newly recorded in Prince Edward Island.

## Introduction

The collection of maple sap for the production of maple sugar has a long history in North America. Before the arrival of European settlers, native people in northeastern North America collected maple sap, pouring it into hollowed-out logs in which heated stones were placed to evaporate the water and concentrate the syrup. In the 1880’s a significant innovation was the introduction of the forerunner to the contemporary flue pan evaporator (Chapeske and Henderson 2007). Two species of trees, sugar maple (Acer saccharum Marsh.) and black maple (Acer nigrum Michx.) are employed in maple sugar production, although the former accounts for the majority of trees tapped (Chapeske and Henderson 2007).

Canada produces 85% of the world’s maple syrup; the United States the other 15%. Annual Canadian production in 2007 was 5.235 million gallons of syrup valued at over $168 million CAD. Québec accounts for 91.1% of domestic production followed by New Brunswick (4.5%), Ontario (3.9%), and Nova Scotia (0.5%). Small volumes are also produced in Prince Edward Island. In the United States, production in 2006 was 1.449 million gallons from producers in Vermont (32%), Maine (21%), New York (17%), Wisconsin (7%), Michigan (5%), Ohio (5%), Pennsylvania (5%), New Hampshire (4%), Massachusetts (3%), and Connecticut (0.7%) (Agriculture and Agri-Food Canada 2007).

In 2006 in Canada, 9,731 maple sugar producers had an average per-farm tap number of 3,913 indicating that approximately 38,077,400 trees were being tapped. Two collection methods are used: the traditional bucket system placed on trees, and plastic tube collection. In Ontario 78% of collection is with plastic tubing and 22% is by traditional buckets (Chapeske and Henderson 2007). In Québec 97.7% collection is with plastic tubing, and only 2.3% with traditional buckets (Johannie Coiteux, Federation of Quebec Maple Syrup Producers, pers. comm.). Employing these percentages indicates that in Canada some 1.5 million trees are tapped using traditional buckets.

Despite the long history of maple syrup production, which has evolved to become a significant industry in Canada and the northeastern United States, and the very sizeable number of trees that are tapped over a considerable portion of the continent, there has been remarkably little attention paid to the insects that are attracted to maple sap during extraction and collection. Sap is largely contained with the plastic tubing collection method and except for local spots surrounding the borehole, there is little opportunity for insects to avail themselves of this resource. In traditional bucket collection the opportunity for attracting insects to sap is considerably greater. Maple sap typically consists of 97.5% water, 2.4% sugars (primarily sucrose with small amounts of glucose), and 0.1% minerals (primarily potassium and calcium, with smaller quantities of zinc and manganese, and trace amounts of other minerals). There are also trace amounts of phenolic compounds, primary amines, peptides, amino acids, and other organic compounds. (Ball 2007).

Many species of moths attracted to sugar solutions on trees, indeed “sugaring” for moths is an important collection technique for many species of nocturnal Lepidoptera. In a poetic essay entitled “Sugaring for Moths” in The Moth Book, Holland (1903: 146–150), an important figure in the history of North American lepidopterology, outlined the technique in a lyrical style, now long vanished from entomological literature. Smith (1900) noted the propensity of many moths to be attracted to the sap of trees, particularly that of sugar maples. He highlighted Xylena spp., Eupsilia spp., Metalepsis salicarum (Walker), Orthosia hibisci (Guenee), and Xystopeplus rufago (Hübner) (all Noctuidae) as species particularly attracted to maple sap. Miller (1997) noted that adult moths of four families – Noctuidae, Sphingidae, Geometridae, and Tortricidae – are attracted to natural sap flows.

Amongst Coleoptera, members of Nitidulidae (sap beetles) are well known to be attracted to natural sap flows on a variety of trees. Vogt (1950) documented 33 species of nitidulids at sap flows, primarily on white oak (Quercus alba L.), but also on post oak (Quercus stellata Wang.), chinkapin oak (Quercus muhlenbergia Engelm.), red maple (Acer rubrum L.), and river birch (Betula nigra L.). In addition to these nitidulids, Vogt (1950) also found representatives of Mycetophagidae (2 species), Histeridae (2 species), Carabidae (1 species), Silvanidae (1 species), Laemophloeidae (1 species), Tenebrionidae (1 species), and Nosodendridae (1 species) at such sap flows.

There has been considerable interest in sap flows caused by Yellow-bellied Sapsuckers (Sphyrapicus varius Linnaeus) (Aves: Picidae) and the insects attracted to these. Studies such as Foster and Tate (1966) and Rissler et al. (1995) recorded a large diversity of insects in Lepidoptera, Hymenoptera, Diptera, Coleoptera, Hemiptera, Homoptera, Collembola, and Psocoptera frequenting sapsucker induced flows. Unfortunately, in many instances identifications were done only to the level of Family.

There are few references in entomological literature of insects specifically associated with the collection of maple sap. Arnett (2000) noted that Hypogastrura nivicola (Fitch) (Collembola: Poduridae), the common and familiar “snow flea”, is attracted to maple sap and can attain pest status in buckets during periods of harvest. Rings (1969, 1973) drew attention to Lithophane antennata (Walker), Lithophane laticinerea Grote, and Lithophane unimoda (Lintner) (Lepidoptera: Noctuidae) as moths attracted to maple sap, which can become a nuisance by accumulating on the surface in collection buckets. Dearborne (1999) reported that adult Ellychnia corrusca (Linnaeus) (Coleoptera: Lampyridae) often become a pest by falling into maple sap buckets.

## Methods

On 4 April 2010 while at a maple sugar collection site in Woodville Mills, Prince Edward Island, (46°14.03333'N, 062°31.06667'W), insects in maple sap collection buckets (n=70) were surveyed. The forest stand (4.2 hectares) consists of primarily deciduous trees [sugar maple, red oak (Quercus rubra L.), yellow birch (Betula alleghaniensis Britt), linden (Tilia europea L), pin cherry (Prunus pensylvanica L.f.), and others) with occasional intermixed conifers [red spruce (Picea rubens Sarg,), white spruce (Picea glauca (Moench) Voss), balsam fir (Abies balsamaea (L.) Mill)]. It is bounded along its lower margin by a small stream flowing from a marsh and beaver pond, along its upper margin by an apple orchard, and along both sides by agricultural fields. The spacing of sap collection buckets varied within this area. In some instance as many as three buckets were place on a single tree; in other instances buckets were as much as 10 meters apart, depending on the size and spacing of the sugar maple trees.

All buckets had covers on them to prevent extraneous debris from falling into the maple sap. Therefore insects present inside would almost certainly have had to actively enter the buckets, either from beneath the lids (where there was a gap at the front of the bucket) or in the small open area near the spigot. Thus, the suite of insects present would largely represent species actively attracted to maple sap, or seeking shelter in the buckets, as opposed to specimens that had accidentally fallen into the containers.

It was not possible to strictly quantify the results, since different buckets had been hanging with uncollected sap for varying periods (1–4 days) due to the impending conclusion of the sap collection season. Nevertheless general categories of abundance [scarce, fewer than 10 specimens; abundant, 10–30 specimens; very abundant, more than 30 specimens] were assigned for each of the species found.

## Results and discussion

The results of this investigation are shown in Table 1. Diptera were also present but were not collected. Twenty-eight species were recorded including 18 Coleoptera, eight Lepidoptera, and two Trichoptera. After reviewing the biology of each species, it was possible to categorize them as species associated with sap and nectar, or accidental visitors.

**Table 1:** Insects collected in maple sap, Woodville Mills, PEI, April 2010

Species	Abundance	PEI Status^1^
COLEOPTERA		
Carabidae		
Dromius piceus Dejean	scarce
Coccinellidae		
Anatis mali (Say)	scarce
Corylophidae		
Orthoperus suturalis LeConte	scarce
Curculionidae		
Trypodendron retusum (LeConte)	abundant	new in PEI
Xyloterinus politus (Say)	scarce
Xyloborinus alni (Niisima) †	scarce	new in PEI
Dermestidae		
Anthrenus castanae Melsheimer	scarce
Histeridae		
Euspilotus assimilis (Paykull)	scarce
Lampyridae		
Ellychnia corrusca (Linnaeus)	very abundant	new in PEI
Nitidulidae		
Cryptarcha ampla Erichson	scarce	new in PEI
Glischrochilus fasciatus (Olivier)	abundant
Glischrochilus quadrisignatus (Say)	abundant
Glischrochilus sanguinolentus (Olivier)	scarce
Glischrochilus siepmanni Brown	scarce
Scirtidae		
Cyphon confusus Brown	scarce	new in PEI
Cyphon variabilis (Thunberg) *	very abundant	new in PEI
Staphylinidae		
Silusa californica Bernhauer	scarce	new in PEI
Nudobius cephalus (Say)	scarce	new in PEI
LEPIDOPTERA		
Noctuidae		
Crocigrapha normani (Grote)	scarce	new in PEI
Eupsilia vinulenta (Grote)	abundant	new in PEI
Eupsilia tristigmata (Grote)	abundant	new in PEI
Lithophane innominata (J.B. Smith)	abundant	new in PEI
Lithophane petulca Grote	scarce	new in PEI
Lithophane pexata Grote	scarce	new in PEI
Xylena cineritia (Grote)	scarce	new in PEI
Tortricidae		
Acleris chalybeana (C.H. Fernald)	abundant	new in PEI
TRICHOPTERA		
Limnephilidae		
Glyphopsyche irrorata (Fabricius)	scarce	new in PEI
Limnephilus ornatus Banks	scarce	new in PEI

†, adventive Palaearctic species; *, Holarctic species. 1 No entry indicates the species has previously been recorded in the province.

Due in large measure to the comparative dearth of entomological research on Prince Edward Island, it is possible to report that 18 of the 28 species found in this study are newly recorded in the province. In the case of some species of Coleoptera (i.e., Ellychnia corrusca, Cyphon variabilis, Cyphon confusus, and Nudobius cephalus (Say)) these new records represent broadly distributed species that belong to groups that have not yet been surveyed on Prince Edward Island. However the Prince Edward Island Curculionidae were surveyed by Majka et al. (2007), the Nitidulidae were surveyed by Majka and Cline (2006a), and the Maritime Provinces Aleocharinae by Majka and Klimaszewski (2010) so the new records of Trypodendron retusum (LeConte), Xyloborinus alni (Niisima), Cryptarcha ampla Erichson and Silusa californica Bernhauer are additions to a fauna which has already received recent attention.

Similarly, although some research has been conducted on the Noctuidae of Prince Edward Island, the fauna remains poorly known and none of the species recorded herein have previously been recorded from the province (Troubridge and Lafontaine 2004).

## Coleoptera: species associated with sap and nectar

Two species were hyper-abundant (more than 100 individuals each) in the sap buckets: Ellychnia corrusca and Cyphon variabilis. Rooney and Lewis (2000) reported that adult Ellychnia corrusca feed actively on floral nectarines of Norway maple (Acer platanoides L.) and are attracted to natural sap flows on Acer saccharum. As previously noted, Dearborne (1999) remarked on how this species was attracted to maple sap, often becoming a pest in sap buckets.

Klausnitzer (2009) described Cyphon variabilis as a eurotypic species found in moderately acid Sphagnum moorlands, near eutrophic waters, beside muddy ditches, and along the banks of ponds. The species has not been generally associated with sap flows or the collection of maple sap in the literature, although Wolcott and Montgomery (1933) did note a specimen on a tamarack trunk, “feeding at small spot of exuding sap.” The collection site is approximately 0.25 km from a small beaver pond and associated marsh that provide a suitable site for Cyphon variabilis, however, the large number of specimens attracted to maple sap (and smaller numbers of the related Cyphon confusus Brown) is unexpected, apparently reflecting an aspect of the biology of these marsh beetles not hitherto noted in the literature.

Trypodendron retusum, Xyloterinus politus (Say), and Xyloborinus alni are all ambrosia beetles (Curculionidae: Scolytinae) wherein adults excavate galleries beneath bark of unhealthy or dying trees. These galleries are inoculated with symbiotic fungi carried in mycangial pits on the heads of the beetle. Adults and larvae feed on the resulting growth of fungal hyphae. Bark beetles are known to be attracted to tree volatiles such as terpenes and oleoresins, so it may be that these species are attracted to the maples via these chemicals signals, and in seeking their source, the beetles secondarily fall into the maple sap. Trypodendron retusum is associated with poplars (Populus grandidentata Michx. and Populus tremuloides Michx.) and is newly recorded on Prince Edward Island (Wood 1982; Majka et al. 2007). Xyloterinus politus is associated with a wide variety of primarily deciduous hosts (occasionally coniferous ones) including species of Acer, Alnus, Betula, Carya, Castanea, Fagus, Fraxinus, Quercus, Picea, Pinus, Tsuga and Ulmus (Wood 1982). Xyloborinus alni is an adventive Oriental species recently found in North America (Haack 2006). The species was originally detected in 1995 in British Columbia, and 1996 in Washington State, and later reported in several eastern states in the USA (Haack 2006). This is the first report of this species from Prince Edward Island. In North America the only reported hosts are Alnus spp. (Haack 2006).

Cryptarcha ampla, Glischrochilus fasciatus (Olivier), Glischrochilus quadrisignatus (Say), Glischrochilus sanguinolentus (Olivier), and Glischrochilus siepmanni Brown are all sap beetles (Nitidulidae: Cryptarchinae) well known to be attracted to a variety of saps and liquids. Vogt (1950) recorded Cryptarcha ampla, Glischrochilus fasciatus, and Glischrochilus quadrisignatus from sap flows on maples. Parsons (1943) also noted that Cryptarcha ampla occurs on sap flows on maple. Williams et al. (1992) collected large numbers of all five of these species from a variety of baits including fermenting bread dough, a fermenting brown sugar solution, and decaying cantaloupes and bananas. Majka and Cline (2006a) noted that in the Maritime Provinces Glischrochilus sanguinolentus was primarily found associated with coniferous trees (Pinus and Picea spp.) but was also occasionally found on sap flows on trembling aspen (Populus tremuloides) and red oak (Quercus rubra L.).

Although Anthrenus larvae such as Anthrenus castanae Melsheimer, like other dermestids, feed on dried animal and plants products, adults mate in the field and feed on nectar and pollen (Bousquet 1990). Consequently, like other nectarivourous species, the single specimen collected may have been attracted to sugars found in maple sap.

## Coleoptera: accidental species

Dromius piceus Dejean is a nocturnal, arboreal predaceous ground beetle found in deciduous, coniferous and mixed forests (Larochelle and Larivière 2003). One specimen was found, and is likely an accidental collection. Similarly Nudobius cephalus (Say) is a nocturnal, predaceous rove beetle found under the bark of trees feeding on various insects found in such habitats (Smetana 1982). The species is primarily associated with coniferous trees, but is occasionally found on deciduous trees (Acer, Betula, Populus spp.) (Smetana 1982). The single individual found may also have been an accidental collection in the course of nocturnal foraging activities. Anatis mali (Say) is a large lady beetle, frequently associated with conifer trees and an important predator of the balsam twig aphid (Mindarus abietinus Koch) (Bethiaume et al. 2004), and its presence in maple sap is probably accidental. Like other corylophids, adults of Orthoperus suturalis LeConte feed on fungal spores and are found in decompositional environments. Majka and Cline (2006b) reported it from Sphagnum bogs and red spruce (Picea rubens Sarg.) forests. Therefore its presence in maple sap is probably accidental. The histerid Euspilotus assimilis (Paykull) is commonly found on carrion (Bousquet and Laplante 2006). Its presence in maple sap is also probably accidental. Silusa californica is a widely distributed boreal rove beetle found in forest litter, wet moss, on dung and fungi in coniferous, deciduous, and mixed forests (Klimaszewski et al. 2003). Its presence in maple sap is also probably accidental.

## Lepidoptera: species associated with sap and nectar

Xylena cineritia (Grote), Lithophane innominata (J.B. Smith), Lithophane petulca Grote, Lithophane pexata Grote, Eupsilia vinulenta (Grote), and Eupsilia tristigmata (Grote) (Noctuiodae: Cuculliinae: Xylenini) were well represented in the maple sap buckets. They are all members of genera well known to be attracted to maple sap (Rings 1969, 1973; Smith 1900). The physiology and behaviour of moths in these genera (specifically Lithophane innominata, Eupsilia tristigmata and Eupsilia vinulenta), all of which are active during the winter months whenever ambient temperatures rise above 0°C, was thoroughly investigated by Heinrich (1987). He found that they are active at low temperatures because they maintain thoracic temperatures 10°C higher than other moths. This is accomplished through a combination of behavioural adaptations (shivering to warm the thorax, which can commence at temperatures of -2°C, much lower than in other Lepidoptera) and anatomical features (a thick pile on the head and thorax, a series of abdominal air sacks that act as insulators, and an aortic configuration that acts as a thoracic heat exchanger). The moths obtain the energy for these physiological processes and activities by utilizing sugar saps. Heinrich (1987) observed that, given the opportunity, these moths will bloat themselves by consuming saps, increasing their body weight by up to 94.5%.

Heinrich (1987) used sugar solutions containing approximately 10 times as much sugar as found in maple sap, and calculated that a meal of this kind contained energy reserves that would last a single moth 31 days. Presumably, a meal of maple sap, containing 10% of the concentration employed by Heinrich (1987), would yield a moth approximately 3 days of energy reserves. Thus, the presence of a diversity of species and substantial numbers of moths in the genera Eupsilia, Lithophane, and Xylena in maple sap buckets is not unexpected.

In addition to the noctuids discussed above, one tortricid, Acleris chalybeana (C.H. Fernald), was abundant in maple sap buckets. This is a widespread species whose hosts include apple, beech, birch, maple, and oak (Covell 1984). Tortricids are one of the four families of moths noted by Miller (1997) and Foster and Tate (1966) that are attracted to natural sap flows. Acleris chalybeana is known to defoliate sugar maple under certain circumstances (Horsley et al. 2002; Hallett et al. 2006). A specific attraction of this species to maple sap, and what role it may play in the physiology of the moth, have not been documented.

## Lepidoptera: accidental species

The one noctuid collected which is not a member of this suite of moths, was a single specimen of Crocigrapha normani (Grote), a species whose hosts include apple, cherry, oak, and other deciduous trees (Covell 1984). It would appear that its presence in the maple sap buckets was accidental.

## Trichoptera

Almost all adult Trichoptera are liquid feeders, consuming sap and floral nectar (Malicky 2004). Single individuals of two species of caddisflies, Glyphopsyche irrorata (Fabricius) and Limnephilus ornatus Banks, were found in the maple sap buckets. Glyphopsyche irrorata is a caddisfly which over-winters as an adult and, like moths of the genera Eupsilia, Lithophane, and Xylena, is regularly active in the winter months at temperatures near 0°C (Berté and Prichard 1983; South 1983).

## Conclusion

In summary, 28 species of Coleoptera, Lepidoptera, and Trichoptera were recovered from maple sap buckets at one site in Prince Edward Island. Nineteen of these are known to be attracted to sap and nectar. Two species, Cyphon variabilis and Cyphon confusus have not been documented as exhibiting an association with such substances, but the hyper abundance of the former species in collection vessels appears to indicate that an association does exist and reflects a hitherto undocumented feature of the biology of these species of marsh beetles. The remaining seven species (all represented by single individuals) are mostly found in deciduous forest stands, and their presence in maple sap containers would appear to be accidental.

Given that some 1.5 million are tapped in Canada employing traditional buckets, the number of insects collected annually through such activities must be considerable. This phenomenon may have both ecological repercussions, in terms of the impact of removing a sizeable number of adults from the population early in the reproductive season, as well as an economic impact in terms of the removal of drowned insects in the maple sap before processing. This preliminary study suggests further research on this phenomenon is needed to document not only a more complete taxon list, but also to aid the maple syrup industry in controlling unwanted insect contaminants through methods utilizing the biology of the species.
